# Putting radioactive materials on the sustainability agenda: a report from a workshop on the sustainability of human-made radioactive materials held at the safeND Research Symposium 2023

**DOI:** 10.1007/s00411-024-01061-8

**Published:** 2024-02-17

**Authors:** Fanny Böse, Alexander Wimmers, Julia Neugebauer, Theresa Lösel, Timo Hermes, Jasmin Beppler, Marie-Sophie Nickel, Pauline Morawe, Maximilian Weber, Christian von Hirschhausen

**Affiliations:** 1 Federal Office for the Safety of Nuclear Waste Management (BASE), Wegelystraße 8, 10623 Berlin, Germany; 2 Workgroup for Infrastructure Policy (WIP), Technical University of Berlin (TU Berlin), Straße des 17. Juni 135, 10623 Berlin, Germany; 3 German Institute for Economic Research (DIW Berlin), Mohrenstraße 58, 10117 Berlin, Germany; 4 TU Berlin, Straße des 17. Juni 135, 10623 Berlin, Germany

**Keywords:** Radioactive waste, Radioactive material, Nuclear waste, Sustainable development goals, Workshop report, Planetary boundaries

## Abstract

This report summarizes the findings of a workshop held at the safeND Research Symposium and hosted by the German Federal Office for the Safety of Radioactive Waste Management (BASE) in Berlin in September 2023. The workshop aimed to channel perspectives from various fields of expertise to discuss key sustainability concepts in terms of radioactive waste management. Therefore, the report highlights that current sustainability concepts, such as the United Nations’ Sustainable Development Goals (SDG) as well as the concept of Planetary Boundaries, neglect challenges arising from the production and storage of human-made radioactive materials. The workshop consisted of three group tasks. The first attempted at identifying the interrelations between “sustainability” and radioactive waste management. The second was to map the global nature of the challenges. The third took first steps to determine a human-made radioactive material as a potential planetary sub-boundary for “novel entities”. All three groups identified valuable knowledge gaps that should be addressed by future research and concluded that radioactive waste management is underrepresented in these sustainability concepts.

## Introduction

The United Nations’ (UN) Sustainable Development Goals (SDGs) were introduced in 2015 aiming at, among other goals, a reduction of global inequality and, environmental pollution, to create a more sustainable future for all of humanity until 2030 (UN 2015, 2023). However, challenges and risks associated with radiation and the management of radioactive materials are so far not explicitly focused upon in this concept. Thus, following the call by Rühm et al. (2023), this report informs of first results from our workshop “Putting Nuclear Waste on the Sustainability Agenda—Integration Into the Concept of Planetary Boundaries” that was held at the safeND Research Symposium on 15 September 2023 in Berlin, Germany.

SafeND is an international conference on the safety of nuclear waste management hosted by the German Federal Office for the Safety of Nuclear Waste Management (BASE) every other year. The conference allows scientists from a wide range of disciplines to present their research findings on the safe handling and disposal of radioactive waste, including both intra- and interdisciplinary discussions. The aim is also to identify research gaps.^1^ The conference brought together a variety of stakeholders from industry, regulation, policymaking, academia, and the civil society in an open and constructive dialog. Interdisciplinary workshops are an important element of the conference, and our workshop “Putting Nuclear Waste on the Sustainability Agenda” was attended by about 25 persons who actively participated as three smaller groups. We offer the participants to continue the exchange on this important topic.

The remainder of this report is structured as follows. First, in the section “The issues at stake”, we introduce the sustainability concepts that were discussed in the above-mentioned workshop and highlight the lack of consideration of radioactive materials therein. Then, in the section “Group work on key questions”, we explain and discuss the group work that was conducted. The section “Conclusion and outlook” closes with a brief conclusion and highlights the need for future research regarding the inclusion of radiation and the management of radioactive materials into sustainability concepts.

## The issues at stake

Today, the world faces diverse and complex challenges, such as poverty, inequalities, global warming, and increasing conflicts—challenges that the UN’s SDGs aim to combat by 2030 (UN 2015, 2023). This particular concept however, has so far paid little attention to issues concerning human-made radiation sources such as radioactive waste from nuclear power plant operations, although the outcomes of former UN conferences, in particular the Agenda 21 of 1992, had explicitly done so (UN 1992). We thus welcome the initiative of the Radiation and Environmental Biophysics journal to call for radiation-related papers that are relevant to the UN SDGs (Rühm et al. 2023).

Going further, achieving the SDGs must be conducted within the capacities of the Earth, represented by the concept of planetary boundaries (Rockström et al. 2009a). This concept is essential to understand interconnected global processes but has so far, similarly to the SDGs, paid little attention to challenges arising from radioactive waste management and ionizing radiation in general (Rockström et al. 2009a, b; Steffen et al. 2015; Persson et al. 2022; Richardson et al. 2023). Rockström et al. (2009a, p. 472) proposed nine so-called planetary boundaries that define “the safe operating space for humanity”. These boundaries are linked to global biophysical system processes that are vital for the preservation of the desirable and stable Holocene state. Examples of these processes are climate change, ocean acidification, or biosphere integrity. The transgression of any of these boundaries induces the shift from a “safe operating space” to a “zone of increasing risk” or even “high risk”. Both of these so-called “risk states” coincide with the potential crossing of thresholds, which could lead to non-linear and irreversible consequences for Earth systems, and thus humanity, on a global scale. These thresholds are also referred to as “tipping points”, an example of which is the loss of the Greenland ice shield. Richardson et al. (2023) find that six out of nine boundaries have left the “safe operating space” and have moved into either an area of increasing risk, e.g., the boundary “fresh water change”, or have transgressed into a high risk zone, e.g., the boundary “biosphere integrity”.

A special case for planetary boundaries is the category of “novel entities”. This boundary constitutes a heterogeneous group of human-made substances for which Earth system impacts remain largely unknown, including human-made radioactive material, in particular radioactive waste and nuclear weapons (Richardson et al. 2023). Thus, Rockström et al. (2009b) suggest the development of several sub-boundaries for “novel entities”. However, radioactive material has not garnered increased attention. Instead, the emphasis has predominantly been on other “novel entities”, in particular plastics (Persson et al. 2022). In contrast, discussions on sustainability and nuclear power have highlighted the low-carbon nature of energy production, while the current volumes of radioactive waste as well as their potential impacts on Earth systems seem to be of a background nature. However, as nuclear power is garnering more and more interest as a potential technology for climate change mitigation via wide-spread energy system decarbonization, these issues must be more prominently addressed (MIT 2018; Cometto et al. 2022; Böse et al. 2024). If more nuclear capacity would be added to decarbonize energy systems, it would even further accumulate radioactive materials, thus increasing pressure on minimizing its release into the environment due to potentially raised risk of release.

Due to its relevance for future research in various disciplines concerned with sustainability, this discrepancy led us to pose the question whether, and if so, how, radioactive waste could be integrated into the above-mentioned sustainability concepts. In this regard, efforts were taken to integrate this research topic into the course “Sustainability Lab” held at the Technical University of Berlin, which provides students of various backgrounds with the opportunity to work on projects for several months. The goal is to publish inter- and transdisciplinary output material.^2^ Therefore, in Spring of 2023, a group of students undertook first steps to analyze how the challenges of radioactive waste management and radiation protection might be integrated into both the SDG and planetary boundary concepts.

The students’ activities supported the preparation of and participation in the above-mentioned workshop at the safeND Research Symposium 2023. The workshop aimed at bringing together a wide range of experts, stakeholders, and interested parties in the area of radioactive waste management to discuss the emerging topics of sustainability in this regard. Three topical discussion themes guided the workshop. First, the investigation of participants’ views from various backgrounds on sustainable development in the context of radioactive waste management. Second, the attempt to establish a global perspective to the challenge of radioactive waste and material management, and third, the cautious attempt to analyze the novel entities boundary from the perspective of radioactive waste management.

Participants were asked to focus their discussions on issues evolving around high-level radioactive waste (HLW), stemming mainly from the operation of nuclear power plants. Future workshop formats and research could include other waste types (intermediate-, low- and very-low-level wastes) from medical applications or uranium mining activities (Yim 2022). Workshop participants engaged in one of the three topical discussions that we summarize in the following section and presented the results to the plenum.^3^

## Group work on key questions

### How do sustainable development and radiation management coincide?

The results of the first group showed that sustainable development in terms of radioactive waste management has various perspectives. First, it was stressed that “sustainability” in terms of radiation management could only be achieved while simultaneously ensuring safety and security of HLW storage. The participants agreed that the current system of interim waste storage and treatment of radioactive waste should be maintained and expanded to deal with increasing waste volumes. This would ensure the minimal release of ionizing radiation into the environment. It was agreed that an upper limit to global radioactive waste accumulation exists which, if exceeded, could lead to the failure of safety and security measures and consequently to the escape of ionizing radiation into the environment. Second, it was suggested that radioactive waste can be categorized as an externality that should be internalized.^4^ In this regard, social components such as acceptability and intergenerational equity should be considered, in addition to technical and economic challenges. Third, it was noted that the management of long-lived radioactive isotopes constituted a long-term burden to society that could be reduced only by the limitation of global radioactive waste accumulation, and the final disposal in deep geological repositories. Hereby, it was stressed that the fragmented composition of very-low-level waste, produced in, e.g., uranium mines, complicated the adequate management thereof, even in highly industrialized countries such as Germany. Instead, this type of waste would necessitate close monitoring and long-term treatment. Overall, the group noted several aspects that should be considered when defining a comprehensive and conclusive definition of sustainability in the context of radioactive waste management. We find this to be an important next step regarding the implementation of radioactive waste management into sustainability concepts.

Discussions also led to the identification of future research questions: Can the amount and composition of currently stored HLW and such that will be produced in the future be accurately determined despite lack of data in some regions of the world and should there be some form of independent global monitoring of these materials? How should other radioactive waste types be included into these discussions? Could a global radioactive waste agreement become feasible, such as is currently being developed for microplastics by the UNEP Program on “Intergovernmental Negotiating Committee on Plastic Pollution” (UNEP 2022)? What are the implications for the social pillar of sustainability of the long-term commitments of managing radioactive waste?

### Can radioactive material be considered a challenge of global significance?

**Fig. 1:**
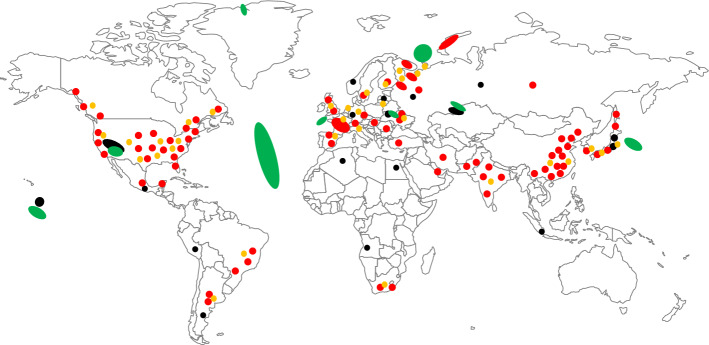
Digitized workshop output on the global significance of radioactive material. Color scheme: Black: Enrichment, fuel production, research, and nuclear explosions; Red: Nuclear plants and other operating facilities; orange: interim storage sites; green: contaminated areas (colour figure online)

The challenge of the second group was to map the global spread of highly radioactive material. To set the scene, participants were given an empty map of the world and asked to highlight regions of heightened occurrence of radioactive material while focusing on HLW production and storage, but also include already contaminated areas. The group then used a color code for different types of radioactive material accumulation to distinguish, for example, waste disposal and storage sites, production facilities, such as nuclear power plants, and then additionally to highlight areas of contamination from nuclear accidents, weapons tests, and waste dumping. Despite limiting the scope to HLW, participants included contaminated areas for which there was uncertainty regarding the level of contamination, e.g., in the Atlantic Ocean (see, e.g., Povinec et al. (2001)). It is important to highlight the explorative nature of this exercise and that participants had no access to literature during the workshop to deny or confirm their assumptions. The result of the group work is shown in Fig. 1.

Participants agreed that highly radioactive material is spread globally. They concluded that most material was concentrated in North America, Europe, Russia, and increasingly Asia, while Africa and Australia remained mostly free from highly radioactive materials and contamination. Some participants suggested to also include low- and intermediate-level material into future applications of this exercise and possibly future research as both Africa and Australia were undoubtedly affected by, for example, uranium mining activities. Other open questions addressed if there were precise quantification and data sources for contaminated sites in particular, and whether the establishment of a global publicly accessible database was feasible. Future research should focus on creating a comprehensive global map of artificially produced radioactive materials, encompassing both stored and released substances.

### How is radioactive waste management related to the “planetary boundaries”?

Following Richardson et al. (2023), radioactive materials are defined as part of the novel entities boundary without further explicit definition. For the sake of this workshop, we limited the scope of these materials to HLW, and asked the participants to discuss the nature of a hypothetical sub-boundary for radioactive materials in the novel entities boundary and if humanity may have already transgressed a "safe operating space" or from which point onwards will have done so.

**Fig. 2:**
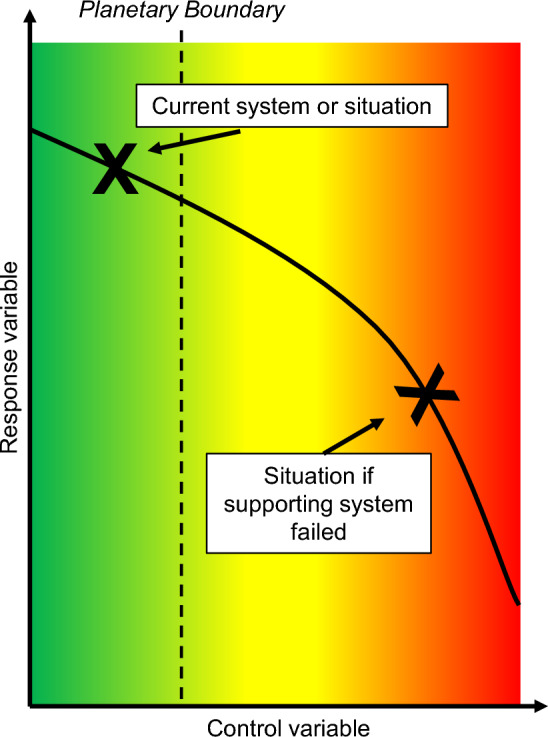
Hypothetical sub-boundary determination of radioactive waste as part of the “Novel entity” boundary. Source: Own depiction based on Rockström et al. (2009b). Color scheme: Green: safe operating space; yellow: area of increasing risk; red: area of (high)—risk (colour figure online)

The group discussion explored the interconnection between safety and security systems of radioactive waste management and the accumulation of HLW. Figure 2 shows the two marks set by the group that visualize two possible states depending on the global state of HLW accumulation and management and the current future trends thereof. Some participants proposed that the current system of interim storage had a low risk to destabilize Earth systems, and thus, as long as safety and security were guaranteed, the current state of waste storage would ensure the safe operating space would not be transgressed. This is shown by the left mark and assumes that current organizational, regulatory, and technical systems are adequate to cope with increasing waste volumes. In contrast, the right mark shows a transgression of the safe operating space that would occur if these safety and security systems were to be overloaded and systems failed. However, the term “overload” was not specified.

Some participants also suggested that the existence of some 495,000 tons of highly radioactive waste, rising annually, already constituted a challenge of global scale, e.g., in the case of war and possible attacks on storage sites. Participants noted that more and more resources would be needed to sustain current safety and security levels, especially if waste accumulation were to continue. These resources themselves were constrained by other planetary boundaries, such as climate change. The transgression of other planetary boundaries could in turn also increase pressure on this system. It was noted that the major challenge in sub-boundary definition would lie in the interpretation of risk. Furthermore, challenges were pointed out, such as the classification of radioactive material as waste, and the necessity to consider all categories of human-made radioactive materials (such as low-, intermediate-, and high-level waste, and all long- and short-lived variations).

During the group discussions, individuals expressed their concern whether radioactive materials were actually an issue of global scale or should instead be regarded as regional challenges. Discussions centered around the question whether plans for deep geological repositories—however slow they might be—per definition reduced the pressure on the current storage systems and thus limited the risk of transgressing a safe operating space. Further questions arose on how a potential control variable to measure the current level of transgression could be defined and monitored. Finally, it was noted that following Richardson et al. (2023, p. 7), as long as material was not “certified as harmless and [was] monitored”, a sub-boundary in the novel entities boundaries should be fixed on zero release to the environment. The final question that remained unanswered was whether this was applicable to radioactive materials today.

## Conclusion and outlook

The workshop showed that “sustainability” in the context of radioactive waste management can be viewed from different perspectives, which necessitates further elaboration for a more comprehensive definition. Such a definition could support the setting of goals and indicators for the UN’s SDGs, or a continuation beyond 2030, and help to determine a safe operating space for humanity. There was a broad consensus among workshop participants that the issue merits further research and debate. Furthermore, all participants agreed that radioactive material management is underrepresented in discussions on sustainability.

Upcoming research should thus address the interrelation between the management of radioactive materials and the SDG framework and whether an inclusion into a post-SDG setting beyond the year 2030 is possible. In addition, research should continue working on the establishment of control variables and the definition of a safe operating space for radioactive materials in the planetary boundary of novel entities.

## Footnotes

1 For more information, refer to https://www.base.bund.de/EN/topics/research/events/safend/safend-2023.html;jsessionid=5EECFA6ACF60CCD87ECB10887054AF01.internet982, accessed 18–10-2023.

2 In German, the course is called “Nachhaltigkeitswerkstatt” (see https://www.tu.berlin/wip/studium-lehre/lehrveranstaltungen, accessed 25–09-2023). The 2022 course, for example, published a book on the history of German nuclear power plants (Camurdas et al. 2023).

3 The workshop was prepared and conducted by the authors of this report. The report was consulted with participants and includes their input. As usual, only the authors are responsible for the final content of this report.

4 In economic theory, externalities occur when decisions of an actor affect the welfare of others (Chavas 2022). This relates to, for example, pollution from automobiles having negative effects on pedestrians that is not adequately accounted for, e.g., via taxes. For radioactive waste, externalities could include the necessity of future generations to deal with the consequences of today’s waste accumulation from energy production in nuclear power plants despite gaining no own welfare increase. Refer to Schulze et al. (1981) for a more detailed account.
